# Selective Inhibition of Phosphodiesterase 7 Enzymes Reduces Motivation for Nicotine Use through Modulation of Mesolimbic Dopaminergic Transmission

**DOI:** 10.1523/JNEUROSCI.3180-20.2021

**Published:** 2021-07-14

**Authors:** Roberto Ciccocioppo, Giordano de Guglielmo, Hong Wu Li, Miriam Melis, Lucia Caffino, Quienwei Shen, Ana Domi, Fabio Fumagalli, Gregory A. Demopulos, George A. Gaitanaris

**Affiliations:** ^1^School of Pharmacy, Pharmacology Unit, University of Camerino, 62032 Camerino, Italy; ^2^Department of Psychiatry, University of California San Diego, La Jolla, California 92093; ^3^Department of Biomedical Sciences, University of Cagliari, 09042 Cagliari, Italy; ^4^Department of Pharmacological and Biomolecular Sciences, University of Milan, 20133 Milan, Italy; ^5^Omeros, Seattle, Washington 98119

**Keywords:** addiction, nicotine, rat, relapse, self-administration, treatment

## Abstract

Approximately 5 million people die from diseases related to nicotine addiction and tobacco use each year. The nicotine-induced increase of corticomesolimbic dopaminergic (DAergic) transmission and hypodopaminergic conditions occurring during abstinence are important for maintaining drug-use habits. We examined the notion of reequilibrating DAergic transmission by inhibiting phosphodiesterase 7 (PDE7), an intracellular enzyme highly expressed in the corticomesolimbic circuitry and responsible for the degradation of cyclic adenosine monophosphate (cAMP), the main second messenger modulated by DA receptor activation. Using selective PDE7 inhibitors, we demonstrated in male rats that systemic PDE7 enzyme inhibition reduced nicotine self-administration and prevented reinstatement to nicotine seeking evoked by cues or by the pharmacological stressor yohimbine. The effect was also observed by direct application of the PDE7 inhibitors into the nucleus accumbens (NAc) shell but not into the core. Inhibition of PDE7 resulted in increased DA- and cAMP-regulated neuronal phosphoprotein and cAMP response element-binding protein and their phosphorylated forms in the NAc. It also enhanced the DA D1 receptor agonism-mediated effects, indicating potentiation of protein kinase A–dependent transmission downstream of D1 receptor activation. In electrophysiological recordings from DA neurons in the lateral posterior ventral tegmental area, the PDE7 inhibitors attenuated the spontaneous activity of DA neurons. This effect was exerted through the potentiation of D1 receptor signaling and the subsequent facilitation of γ-aminobutyric acid transmission. The PDE7 inhibitors did not elicit conditioned place preference and did not induce intravenous self-administration, indicating lack of reinforcing properties. Thus, PDE7 inhibitors have the potential to treat nicotine abuse.

**SIGNIFICANCE STATEMENT** The World Health Organization estimates that there are 1.25 billion smokers worldwide, representing one-third of the global population over the age of 15. Nicotine-induced increase of corticomesolimbic DAergic transmission and hypodopaminergic conditions occurring during abstinence are critical for maintaining drug-use habits. Here, we demonstrate that nicotine consumption and relapse to nicotine seeking are attenuated by reequilibrating DAergic transmission through inhibition of PDE7, an intracellular enzyme responsible for the degradation of cAMP, the main second messenger modulated by DA receptor activation. PDE7 inhibition may represent a novel treatment approach to aid smoking cessation.

## Introduction

The World Health Organization (WHO) estimates that there are 1.25 billion smokers worldwide, representing one-third of the global population over the age of 15 (World Health Organization, 2011). The WHO further estimates that 5 million deaths occur each year as a result of tobacco use. In industrialized countries, ∼90% of lung cancer, 80% of chronic respiratory disease, and ∼20% of cardiovascular diseases are attributed to tobacco use.

Addiction to nicotine is the proximate cause of these diseases (Benowitz, 2010). When administered, nicotine increases the activity of dopaminergic (DAergic) neurons in the ventral tegmental area (VTA) with a subsequent surge of DA release in terminal areas including the nucleus accumbens (NAc) and the prefrontal cortex (PFC; Corrigall et al., 1994; Pontieri et al., 1996; Marti et al., 2011). This is considered the primary mechanism through which nicotine elicits reward and initiates addiction. However, place conditioning studies in rodents suggested that DA transmission is also involved in the modulation of negative states associated with nicotine use (Sellings et al., 2008; Grieder et al., 2012). Following protracted nicotine exposure, the surge of withdrawal and reduction of mesolimbic DA are thought to be important factors for the maintenance of nicotine-taking behavior (Rahman et al., 2004; Bruijnzeel and Markou, 2005; Natividad et al., 2010; Zhang et al., 2012). Rats trained to lever press for nicotine under experimental conditions like those used in the current study have increased extracellular DA levels in the NAc shell following acute intravenous infusion of the drug. However, 24 h after self-administration, extracellular DA concentrations were significantly depressed compared with nicotine naive controls (Rahman et al., 2004). Reduced striatal levels of D1, D2, and D3 receptors, potentially indicating a decrease in DA transmission, have been documented also in abstinent and nonabstinent chronic smokers (Dagher et al., 2001; Yasuno et al., 2007; Fehr et al., 2008). Nicotine exposure has also been shown to alter DA-related intracellular signaling in the NAc and the VTA. In these areas, activation of D1 receptors leads to elevated cyclic adenosine monophosphate (cAMP) levels and enhanced protein kinase A (PKA) signaling, whereas activation of D2 receptors has the opposite effect (Kebabian and Greengard, 1971; Beaulieu and Gainetdinov, 2011). These changes in cAMP activity reflect nicotine's ability to modulate in the NAc and the VTA, in a time- and dose-dependent manner, the expression and phosphorylation of cAMP response element-binding protein (CREB) as well as DA- and cAMP-regulated neuronal phosphoprotein (DARPP-32), two main transcription factors regulated by D1 and D2 receptors. (Pandey et al., 2001; Brunzell et al., 2003; Hamada et al., 2004, 2005; Brunzell and Picciotto, 2009).

Phosphodiesterases (PDEs) are enzymes that regulate intracellular levels of the second messengers cAMP and cGMP by controlling their rates of degradation. Eleven different PDE families have been identified, and each family has several isoforms and splice variants. These unique PDEs differ in their substrate specificities, kinetic properties, modes of regulation, intracellular localization, and cellular expression (Bender and Beavo, 2006). The phosphodiesterase-7 (PDE7) family encompasses two members, PDE7A and PDE7B. Both are cAMP-specific enzymes expressed in areas of the brain linked to addiction. Specifically, PDE7A is highly expressed in the striatum, the substantia nigra, the hippocampus, and the cerebellum (Miró et al., 2001). PDE7B is predominantly expressed in the olfactory tubercle, the NAc, the islands of Calleja, the striatum, and some thalamic nuclei (Reyes-Irisarri et al., 2005; Johansson et al., 2012). Expression of both PDE7A and PDE7B has been detected in the VTA and the PFC.

Considering the expression of PDE7 in reward-related neurocircuitry and the potential role of these enzymes in regulating the DA-cAMP-PKA pathway, we investigated the role of PDE7 in nicotine self-administration and in reinstatement of nicotine seeking. In this article, we describe the effects of selective PDE7 inhibitors (PDE7i) in behavioral, electrophysiological, and biochemical models of nicotine self-administration in the rat.

## Methods and Materials

### Animals

Male Wistar rats (Charles River Laboratories) weighing 150–200 g at the beginning of the experiments were used. Pairs of rats were housed in a room with artificial 12:12 h light/dark cycle (lights off at 9:00 A.M.) at constant temperature (20–22°C) and humidity (45–55%). All training and experimental sessions were conducted during the nocturnal phase of the light/dark cycle.

Animals were given *ad libitum* access to food and water throughout except during experimental test sessions. All the procedures were conducted in adherence with the European Community Council Directive for Care and Use of Laboratory Animals and the National Institutes of Health Guide for the Care and Use of Laboratory Animals.

#### Drugs

Nicotine (Sigma) was dissolved in a sterile saline solution (0.9% NaCl), and the pH was adjusted to 7. The drug was given intravenously, and nicotine self-administration doses are reported as free base concentrations. OMSPDE79 and OMSPDE71 were provided by Omeros. For peripheral administration, compounds were dissolved in 0.03 m tartaric acid in distilled water, the pH was adjusted to 6, and the compounds were delivered intraperitoneally (i.p.). For electrophysiological experiments, OMSPDE79 was dissolved in dimethyl sulfoxide (DMSO) at a final concentration of <0.01%. At the completion of the recording the drug did not wash out. For intracranial injections, compounds were dissolved in a vehicle composed of 20% (v/v) of DMSO, 3% of Tween 80, and 77% of distilled Millipore water. Using a stainless-steel injector, solutions were administered intracranially in a volume of 0.6 µL/rat (0.3 µL/site). Cocaine (Research Triangle Park) was supplied through the drug supply program of the National Institute on Drug Abuse. SKF-82958 and yohimbine (Sigma) were dissolved in 0.9% saline and injected intraperitoneally.

#### Intravenous and intracranial surgeries

Chronic jugular intravenous catheter implantation was conducted as previously described (Ciccocioppo et al., 2001). Briefly, animals were anesthetized by intramuscular injection of 100–150 µL of a solution containing tiletamine chloridrate (58.17 mg/ml) and zolazepam chloridrate (57.5 mg/ml). For intravenous surgery, incisions were made to expose the right jugular vein, and a catheter, made from silicon tubing (inner diameter = 0.020 inches, outer diameter = 0.037 inches), was subcutaneously positioned. After insertion into the vein, the proximal end of the catheter was anchored to the muscles underlying the vein with surgical silk. The distal end of the catheter was attached to a stainless-steel cannula bent at a 90° angle. The cannula was inserted in a support made by dental cement on the scull of the animals, fixed with screws and covered with a plastic cap. For 1 week after surgery, rats were daily treated with 0.2 ml of the antibiotic sodium cefotaxime (262 mg/ml). For the duration of the experiments, catheters were daily flushed with 0.2–0.3 ml of heparinized saline solution. Body weights were monitored every day, and catheter patency was confirmed approximately every 3 d with an injection of 0.2–0.3 ml of thiopental sodium (250 mg/ml) solution. Patency of the catheter was assumed if there was an immediate loss of reflexes. Self-administration experiments began 1 week after surgery.

For the intracranial surgery, the animals underwent stereotaxic surgery in which bilateral cannulae were implanted and aimed at the shell or the core portion of the NAc. Animals were anesthetized by intramuscular injection of 100–150 µL of a solution containing tiletamine chloridrate (58.17 mg/ml) and zolazepam chloridrate (57.5 mg/ml) and placed in a Kopf stereotaxic frame (Kopf Instruments). Guide cannulae were implanted in the NAc with the following coordinates with reference to Bregma: NAc shell, anteroposterior +1.7; lateral ± 1.0; ventral −6.5; NAc core anteroposterior +1.7; lateral ± 1.8; ventral −6.9. Coordinates were taken from Paxinos and Watson (2013) and adjusted for the body weight of the animals. Injectors for drug delivery were 1.5 mm longer than the guide cannula.

The drug was administered using a Hamilton microsyringe in a volume of 0.3 µL/site by means of a stainless-steel injector 1.5 mm longer than the guide cannula so that its tip protruded into the areas. After the experiments, to verify the cannula placement, rats were lightly anaesthetized with isoflurane, and 0.3 µL/site Malachite Green solution was injected into the area. After the animals were killed, the ink diffusion into the region was histologically evaluated.

#### Operant training

The self-administration stations consisted of operant conditioning chambers (Med Associates) enclosed in lit, sound-attenuating, ventilated environmental cubicles. Each chamber was equipped with two retractable levers located in the front panel. Nicotine was delivered by a plastic tube that was connected to the catheter before the beginning of the session. An infusion pump was activated by responses on the right or active lever, and responses on the left or inactive lever were recorded but did not result in any programmed consequences. Activation of the pump resulted in a delivery of 0.1 ml of fluid. An IBM compatible computer controlled the delivery of fluids and the recording of the behavioral data. For the food self-administration study, each chamber was equipped with a food receptacle into which food pellets (45 mg each; Bio-Serv) could be dispensed from a pellet dispenser that was located between the two levers.

#### Nicotine self-administration fixed ratio

Rats were trained to self-administer nicotine in 2 h daily sessions on a fixed ratio 1 (FR1) contingency in which every three lever responses resulted in delivery of a single dose of nicotine (30 µg/0.1 ml). Self-administration training continued until rats showed a stable baseline of reinforced lever pressing. Once the baseline was reached, they were subjected to treatment with PDE7 inhibitors. When intracranial surgery was required, rats were first trained until the nicotine self-administration baseline was established. Rats were then subjected to stereotaxic surgery for cannula placement and allowed to recover for 1 week. After 3–4 d of nicotine self-administration to reestablish the baseline, drug testing began. Operant responses, at both active and inactive levers, were recorded. Drug testing was conducted in a within-subject Latin square design in which at least 2–3 d were allowed between treatments. In the in-between treatment interval, the nicotine baseline was reestablished.

#### Nicotine self-administration progressive ratio

Rats were first trained to FR1 self-administration as previously described. Once a stable baseline was reached, they were switched to a progressive ratio (PR) contingency. Under these conditions, the response requirements necessary to receive a single drug dose increased according to the following equation: [5e ^(injection numbers × 0.2)^] − 5. This resulted in the following progression of response requirements: 1, 2, 4, 6, 9, 12, 15, 20, 25, 32, 40, 50, 62, 77, 95, 118, 145, 178, 219, 268, and so on. The break point was defined as the last ratio attained by the rat before a 60 min period during which a ratio was not completed. Rats were first trained to FR1 nicotine self-administration and then switched to a PR schedule of reinforcement. A 3 d interval was allowed between PR tests during which the animals were subjected to FR1 nicotine self-administration to maintain baseline intake.

#### Cue-induced reinstatement of nicotine seeking

This experiment consisted of the following three phases: discrimination training, extinction, and reinstatement.

##### Discrimination training phase

The purpose of this procedure was to train the rats to self-administer nicotine intravenously while simultaneously establishing discriminative stimuli associated with nicotine availability or nonavailability. Once rats developed stable levels of 2 h daily nicotine (30 µg/infusion) intake, they were subjected to a discrimination-learning regimen. Specifically, in three daily 1 h sessions, either nicotine or saline was available as the only infusion solution. Each training day included two 1 h nicotine sessions and one 2 h saline session in a random order. Between sessions, animals were removed from the operant boxes for at least 1 h. The sessions were initiated by extension of the levers into the chambers and concurrent onset of the respective discriminative stimuli, which remained present until termination of the session by retraction of the levers. The discriminative stimuli associated with the nicotine availability (S+) consisted of an intermittent tone (7 kHz, 70 dB), whereas the discriminative stimuli predictive of the saline vehicle solution (S− or no reward) consisted of continuous illumination of the self-administration chamber's house light. To prevent accidental overdosing, drug infusions were followed by a 20 s time-out (TO) period, which was signaled by illumination of a white cue light while the lever remained inactive. Saline infusions produced by lever presses during the S− sessions were followed by a 20 s TO period and signaled by a white noise (70 dB). In both the S+ and the S− conditions, two levers were present. Only the right lever was active and produced an intravenous infusion of the respective solution when pressed. The left lever was inactive and responses at this lever were recorded as a measure of nonspecific activation.

##### Extinction phase

Responses at the previously active lever activated the syringe pump motor only and had no other programmed consequences (neither nicotine nor saline were administered, and no cues were presented). These sessions lasted 1 h and were conducted once daily until extinction of response was reached (<10 total responses over the last three sessions).

##### Reinstatement test

Reinstatement testing began the day after the last extinction day. To evaluate the effect of PDE7 inhibition on cue-induced nicotine seeking, rats were treated with a compound or vehicle in a Latin square counterbalanced order. The reinstatement test lasted 1 h under conditions identical to those in the discrimination phase, except that nicotine was not available. PDE7 inhibitors were given in the S+ condition, and an interval of three d was allowed between tests.

#### Yohimbine stress-induced nicotine seeking

Rats were trained to self-administer nicotine under an FR1 schedule of reinforcement; each active lever pressing resulted in the delivery of one nicotine dose (30 μg/0.1 ml infusion) for 2 h. Following each nicotine infusion, a 20 s TO period occurred, during which pressing the active lever did not lead to programmed consequences. TO was accompanied by illumination of a cue light located above the active lever to signal delivery of the positive reinforcement while an intermittent tone was sounded throughout the 2 h session. Following acquisition of a stable baseline of intravenous nicotine self-administration, rats were subjected to the extinction phase.

##### Extinction

During this phase, lever presses were no longer contingently associated with nicotine delivery, hence, operant responding rapidly decreased. The day after the last extinction session, rats were subjected to the reinstatement test.

##### Reinstatement

To evaluate the effect of PDE7 inhibition on yohimbine stress-induced reinstatement, two groups of rats were administered compounds in a counterbalanced Latin square design 45 min before the reinstatement test. To elicit reinstatement, yohimbine (1.25 mg/kg) was given to all animals 30 min after administration of the PDE7 inhibitor, which corresponds to 30 min before the start of the reinstatement session. The yohimbine dose, time of injection, and experimental design were as described in previous studies (Marinelli et al., 2007). Reinstatement experiments and drug treatments were performed every third day. Between tests, rats were subjected to extinction sessions.

### Food self-administration and seeking

Rats (*n* = 8) were trained to self-administer food pellets (45 mg) under an FR1 schedule of reinforcement for 30 min per day. Following each food pellet delivery, a 20 s TO period occurred during which responses at the active lever had no programmed consequences. This TO period was concurrent with illumination of a cue light located above the active lever to signal delivery of the positive reinforcement. The rats were trained to self-administer food for several days until a stable baseline (variation <10% over the last three sessions) of reinforcement was established. At this point, an extinction session was introduced during which lever presses were no longer contingently associated with food delivery. Thirty minutes before the extinction session, rats were treated intraperitoneally with OMSPDE71 (9 mg/kg) or its vehicle, and operant responding in the absence of food reinforcement was recorded for 30 min (see Figure 3). Lever presses in the absence of food delivery was considered a measure of seeking response.

#### Locomotor activity

Automated locomotor activity boxes (Med Associate) were used to quantify behavioral activity. Each animal was placed in the activity box, a square plastic box measuring 43 × 43 × 30 cm, and spontaneous locomotor activity parameters were monitored. Activity was recorded for 15 min starting 5 min after placing the animal in the test cage. Locomotor activity of each rat was automatically recorded by interruption of orthogonal light beams. The behavioral parameter observed was locomotion (as reflected by the number of beam breaks). A locomotor activity test was performed during the dark phase of the light/dark cycle.

### Preparation of protein extracts and Western blot analyses

Rats trained to self-administer nicotine in 2 h daily sessions on an FR1 schedule were subjected to treatment with OMSPDE79. Twenty-four hours after the last self-administration session, and 30 min after the inhibitor injection, rats were killed, and the NAc was immediately dissected, frozen on dry ice, and stored at −80°C. NAc was then homogenized in a glass potter using a cold buffer containing 0.32 m sucrose, 1 mm HEPES solution, 0.1 mm EGTA, 0.1 mm phenylmethanesulfonyl fluoride (PMSF), pH 7.4, in presence of a complete set of protease inhibitors and a phosphatase inhibitor cocktail. The homogenized tissues were centrifuged at 1000 × *g* for 10 min; the resulting pellet (P1), corresponding to the nuclear fraction, was resuspended in a buffer (HEPES, 20 mm; dithiothreitol, 0.1 mm; EGTA, 0.1 mm) with protease and phosphatase inhibitors. Total proteins were measured in the nuclear fraction by the Bio-Rad Protein Assay using bovine serum albumin as the calibration standard (Bio-Rad Laboratories). CREB and DARPP32 analyses were performed on the nuclear fraction.

Ten µg of proteins for each sample were run on an SDS-10% polyacrylamide gel under reducing conditions and then electrophoretically transferred onto nitrocellulose membranes (GE Healthcare Systems). Blots were blocked 1 h at room temperature with 10% nonfat dry milk in tris-buffered saline plus 0.1% Tween-20 buffer, incubated with antibodies against the phosphorylated forms of the proteins, and then stripped and reprobed with the antibodies against corresponding total proteins.

The conditions of the primary antibodies were the following: antiphospho-Darpp32 (Thr34, 1:1000; Cell Signaling Technology), antiphospho-CREB (Ser133, 1:1000; Cell Signaling Technology), antitotal-DARPP32 (1:1000; Cell Signaling Technology), antitotal-CREB (1:1000; Cell Signaling Technology), and anti-β-Actin (1:10 000; Sigma-Aldrich). Results were standardized using β-actin as the control protein, which was detected by evaluating the band density at 43 kDa. Immunocomplexes were visualized by chemiluminescence using the Chemidoc MP Imaging System (Bio-Rad Laboratories) and analyzed using the Image Lab software from Bio-Rad.

### Electrophysiology

The preparation of ventral tegmental area (VTA) slices was as described previously (Melis et al., 2010). Briefly, male Sprague Dawley rats (14–22 d) were anesthetized with halothane in a vapor chamber and killed by decapitation. A block of tissue containing the midbrain was rapidly dissected and sliced in the horizontal plane (300 µm) with a vibratome (Leica VT1000 S) in ice-cold low-Ca^2+^ solution containing the following (in mm): 126 NaCl, 1.6 KCl, 1.2 NaH_2_PO_4_, 1.2 MgCl_2_, 0.625 CaCl_2_, 18 NaHCO_3_, and 11 glucose. Slices were transferred to a holding chamber with artificial cerebrospinal fluid (ACSF; 37°C) saturated with 95% O_2_ and 5% CO_2_ containing the following (in mM): 126 NaCl, 1.6 KCl, 1.2 NaH_2_PO_4_, 1.2 MgCl_2_, 2.4 CaCl_2_, 18 NaHCO_3_, and 11 glucose. Slices were allowed to recover for at least 1 h before being placed in the recording chamber and superfused with the ACSF (37°C) saturated with 95% O_2_ and 5% CO_2_. Cells were visualized with an upright microscope with infrared illumination (Axioskop FS 2 plus, Zeiss) and whole-cell patch-clamp recordings were made by using an Axopatch 200B Amplifier (Molecular Devices). Current-clamp experiments were made with electrodes filled with a solution containing the following (in mm): 117 KCl 144, 10 HEPES, 3.45 BAPTA, CaCl_2_ 1, 2.5 Mg_2_ATP, and 0.25 Mg_2_GTP (pH 7.2–7.4, 275–285 mOsm). Experiments were begun only after series resistance had stabilized (typically 15–40 MΩ). Data were filtered at 2 kHz, digitized at 10 kHz, and collected on-line with acquisition software (pClamp version 8.2, Molecular Devices). DA neurons from the lateral portion of the posterior VTA were identified by the presence of a large (>100 pA) I_h_ current that was assayed immediately after break in, using a series of incremental 10 mV hyperpolarizing steps from a holding potential of −70 mV (Johnson and North, 1992). Each slice received only a single drug exposure. Drug-induced changes in the firing rate were calculated by averaging the effects after drug administration (5 min) and normalizing to the predrug baseline. All the numerical data are given as mean ± SEM. Data were compared and analyzed by using two-way ANOVA for repeated measures (treatment × time) or, when appropriate, Student's *t* test for repeated measures. Statistical analysis was performed by means of the GraphPad Prism program. The significance level was established at *p* < 0.05.

### PDE7 inhibitors

The PDE7 inhibitors have been described previously (Inoue et al., 2014). The inhibitory potency (IC_50_ value) and PDE7 selectivity for each compound were determined *in vitro* using a scintillation proximity assay to quantitate the conversion of ^3^[H]-cAMP to ^3^[H]−5′ AMP. Eight dilutions of inhibitor were assayed in 50 mm Tris–HCl pH 7.5, 8.3 mm MgCl2, 0.5 mg/ml BSA, 1.7 mm EGTA, 1% DMSO, and 16 nm ^3^[H]-cAMP. At this substrate concentration, the IC_50_ approximates the inhibition constant. To start the reaction, PDE7A or PDE7B was added to a final concentration of 25 ng/ml. The reaction was incubated at 30°C for 20 min. It was terminated by the addition of yttrium silicate beads (catalog #RPNQ0150, GE Healthcare,) and counted on a Wallac MicroBeta scintillation counter 2 h following the addition of the beads. Samples were run in duplicates. Data were analyzed using XLfit (Microsoft) from which C_50_ values were obtained. In each case, the IC50 was <20 nm.

For the selectivity assays, we used ^3^[H]-cAMP for PDE3, PDE4, and PDE8 and ^3^_H_-cGMP instead of ^3^[H]-cAMP for PDE1, PDE2, PDE5, PDE9, PDE10, and PDE11. We used the human PDEs, as no differences in activity were observed between human and rodent enzymes with several control compounds tested (data not shown). The enzymes were obtained from BPS BioScience. In each case, the selectivity against the other PDEs tested was over at least 100-fold lower (Table 1). It should be noted that OMSPDE79 and OMSPDE71 were also evaluated at 10 μΜ each for interactions with 173 different proteins (Eurofins Scientific). Although the concentrations of the PDE7 inhibitors were 500-fold higher than their PDE7 IC50s, no significant responses (over 50% inhibition) were detected in either case (data not shown).

**Table 1.** Fold selectivity of PDE7 inhibitors against members of the PDE family

PDE	OMSPDE71	OMSPDE79
1B	>1235	>562
2A	1713	1400
3A	>1235	1744
4A	>100	>100
5A	2569	1106
7A	1	1
8A	4104	>562
9A	>1235	>562
10A	212	>562
11A	>1235	>562

To assess the pharmacokinetic properties of the PDE7 inhibitors, Sprague Dawley rats (*n* = 5 or 3, respectively; Charles River Laboratories) were dosed intraperitoneally with a 9 mg/kg (10 ml/kg) compound suspended in 0.03 m tartaric acid. Blood was drawn for isolation of plasma at the following postdose intervals (in hours): 0.25, 0.5, 1, 2, 3, 4, and 24. Compounds were quantitated using a ThermoFisher TSQ Ultra Triple Quadrupole Mass Spectrometer in conjunction with a Thermo Scientific Accela 1250 HPLC Pump. Plasma samples and standards were extracted by protein precipitation with acetonitrile, filtered with a Sirocco protein precipitation plate, dried under nitrogen at 50° C, and then reconstituted with 20/80 acetonitrile/water. The HPLC tandem mass spectrometry method used to quantitate these compounds from plasma used a Waters XBridge C18 column (2.1 × 30 mm) with a Phenomenex SecurityGuard column. The HPLC separation method applied a fast gradient of mobile phase A: 10 mm ammonium formate in 5/95 acetonitrile/water with 0.05% acetic acid, and mobile phase B: 10 mm ammonium formate in 95/5 acetonitrile/water with 0.1% acetic acid. The %A was 100% from *t* = 0–1 min; switched to 100%B from 1.0 to 3.0 min, held at 100%B to 4.5 min, and then reequilibrated to 100%A from 4.5 to 5.5 min. Sample extracts were reconstituted in mobile phase, and 10 µL were injected for electrospray ionization. Multiple reaction monitoring transitions were recorded for each molecule through 1.5 mTorr argon gas between 24 and 30 eV collision energy. The range of quantitation was 0.5–10,000 ng/ml. Plasma half-lives were ascertained from the slope of the respective elimination semilog concentration versus time profiles by the following equation: t1/2 = ln(2)/λz, where λz is the slope. They ranged from 0.7 to 1.3 h. Brain tissue levels were determined by homogenizing the whole rat brain in a 50:50 acetonitrile-to-water solution, followed by protein precipitation, and then filtering with a Sirocco protein precipitation plate. The range of quantitation in rat brain tissue was 5–10,000 ng/g. For the brain-to-plasma (B:P) ratio determination, intravenous infusion and tissue harvests were used. Both compounds crossed the blood–brain barrier.

### Experimental design and statistical analysis

Male Wistar rats were used, and control groups and sample size are indicated below in Results. For behavioral and biochemical experiments using STATISTICA version 7 software, data were analyzed by one-way or two-way ANOVA followed by Dunnett's or Tukey's *post hoc* test, respectively. The significance level was established at *p* < 0.05. For electrophysiology, drug-induced changes in firing rates were calculated by averaging the effects 5 min after drug administration and normalizing to the predrug baseline. All the numerical data are given as mean ± SEM. Data were compared and analyzed using two-way ANOVA for repeated measures (treatment × time) or, when appropriate, by Student's *t* test for repeated measures. The significance level was established at *p* < 0.05.

## Results

### Inhibition of PDE7 attenuates nicotine self-administration

**Figure 1.**
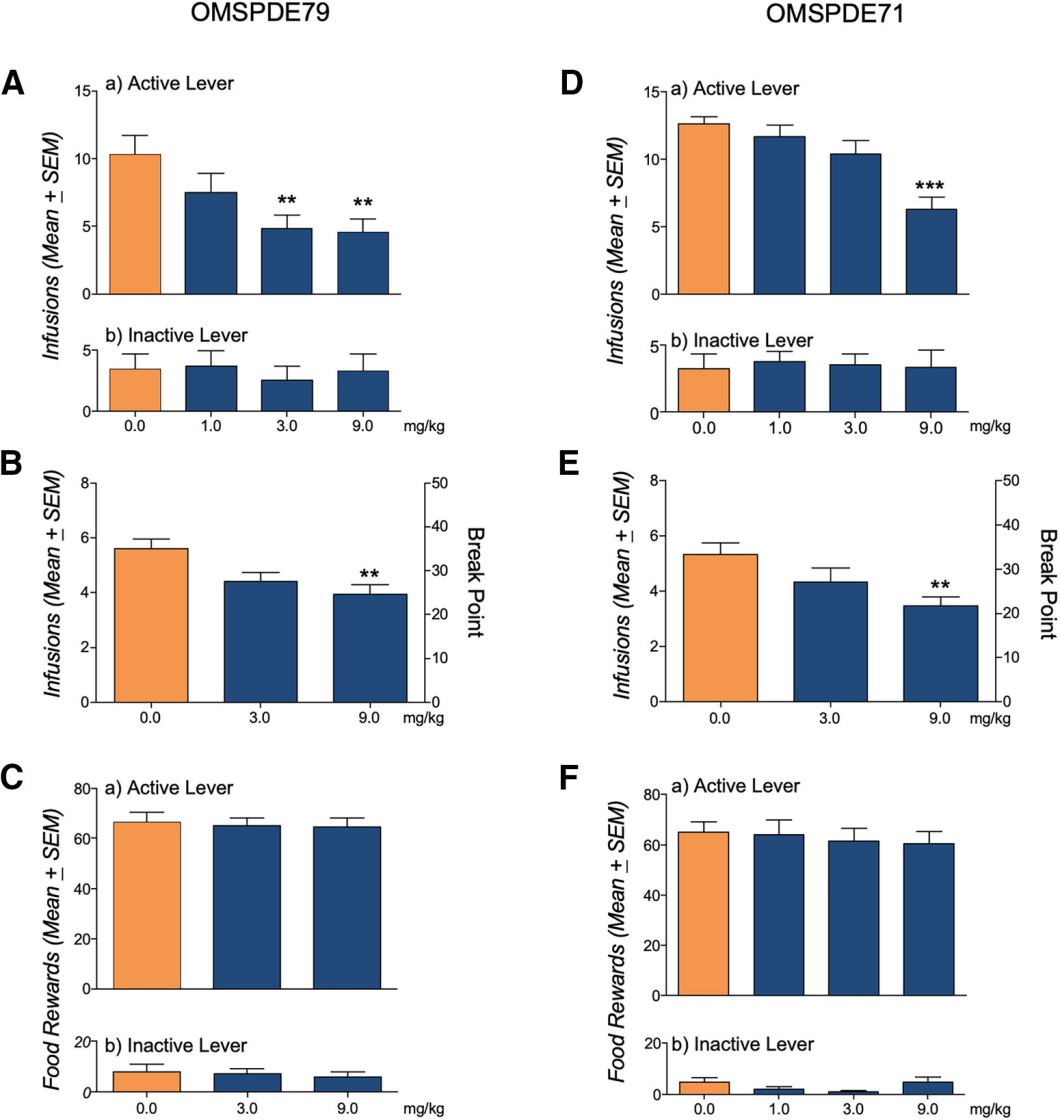
Effect of PDEi on nicotine and on food self-administration. ***A*, *D***, Rats (*n* = 5) were trained to a 2 h FR1 nicotine self-administration until a stable baseline of nicotine infusions was reached. In a counterbalanced Latin square design, rats were then treated intraperitoneally with OMSPDE79 (*n* = 15) or OMSPDE71 (*n* = 12) 30 min before the beginning of the test session. ***B***, ***E***, In rats (*n* = 15 for each PDE7i) trained to a PR schedule of reinforcement, compounds significantly decreased PR responding as revealed by a reduction in nicotine infusions and break point. ***C*, *F***, In rats trained to food self-administration, operant responding was not affected either by OMSPDE79 or OMSPDE71. Values represent the mean ± SEM. Significant difference from vehicle (0.0), **p < 0.01 and ***p < 0.001.

We first evaluated the effects of OMSPDE79 and OMSPDE71 on intravenous nicotine self-administration by training rats to lever press for nicotine (0.03 mg/0.1 ml infusion) under an FR1 schedule of reinforcement. Training continued until rats exhibited a stable level of responding. At this point, two groups of rats underwent intraperitoneal administration of OMSPDE71 (0.0, 1.0, 3.0, and 9.0 mg/kg; *n* = 12) or OMSPDE79 (0.0, 1.0, 3.0, and 9.0 mg/kg; *n* = 15), respectively, 30 min before the beginning of the session. As shown in Figure 1*A*,*D*, treatments significantly decreased operant responding for nicotine: (*F*_(3,14)_ = 6.31, *p* = 0.0062) for OMSPDE79 and (*F*_(3,11)_ = 16.68, *p* = 0.0002) for OMSPDE71. Dunnett's *post hoc* analysis demonstrated a significant reduction of nicotine-reinforced responses at the concentrations of 3.0 and 9.0 mg/kg of OMSPDE79 (**p < 0.01 vs 0.0 mg/kg) and at 9.0 mg/kg for OMSPDE71 (**p < 0.01 vs 0.0 mg/kg). Inactive lever responses were not affected by the treatment [*F*_(3,14)_ = 0.22, *p* = 0.88 (not significant; [NS])] for OMSPDE79 and [*F*_(3,11)_ = 0.43, *p* = 0.74 (NS)] for OMSPDE71. To further assess the ability of PDE7i to attenuate the motivation for nicotine, we tested each compound's effects on nicotine self-administration in new groups of rats (*n* = 15 for each PDE7i) under a PR schedule of reinforcement. As shown in Figure 1*B*,*E*, both compounds significantly decreased PR responding as revealed by reduction in nicotine infusions as follows: (*F*_(2,14)_ = 5.91, *p* = 0.013) for OMSPDE79, (*F*_(2,14)_ = 5.05, *p* = 0.022) for OMSPDE71 and break point (*F*_(2,14)_ = 4.86, *p* = 0.025), and (*F*_(2,14)_ = 3.83, *p* = 0.047) for OMSPDE79 and OMSPDE71, respectively. Compared with the vehicle, Dunnett's *post hoc* analysis demonstrated a significant difference at 9.0 mg/kg (**p < 0.01) of OMSPDE79 and 9.0 mg/kg (*p < 0.05) of OMSPDE71. These effects on nicotine self-administration were selective because, as shown in Figure 1*C*,*F*, the two PDE7 inhibitors did not change the operant response for food in rats (*n* = 8 for each PDE7i) trained to 30 min FR1 food pellets (45 mg) self-administration. In fact, ANOVAS showed no drug effects at the active [*F*_(2,7)_ = 0.16, *p* = 0.85 (NS)] and inactive levers [*F*_(2,7)_ = 0.75, *p* = 0.5 (NS)] for OMSPDE79, or [*F*_(3,7)_ = 0.27, *p* = 0.84 (NS)] at the active lever and [*F*_(3,7)_ = 2.51, *p* = 0.14 (NS)] at the inactive lever for OMSPDE71.

### Nicotine self-administration is attenuated following PDE7 inhibition in the NAc shell but not in the NAc core

**Figure 2.**
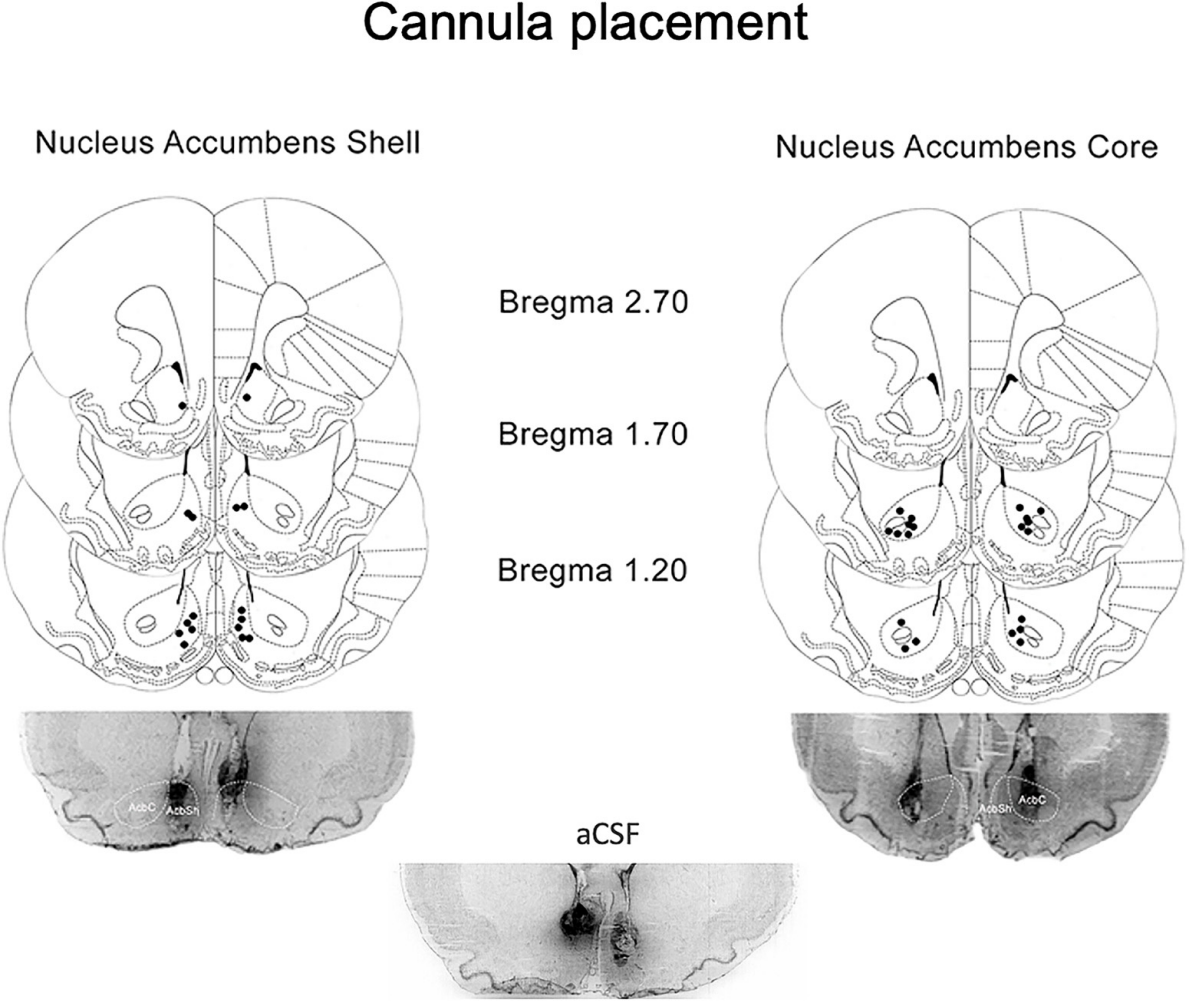
Histologic evaluation of intracranial cannula placement. The injection sites were confirmed for both NAc shell and NAc core. Histologic analysis confirmed bilateral injections into the NAc shell in eight animals and NAc core in 10 animals. These rats were used for the analysis of the effects of microinjection of PDE7 inhibitors into the NAc shell and NAc core. Animals with incorrectly placed cannulae were excluded from analysis. The rats in which the cannulas missed the NAc shell did not respond to the PDE7. PDE7 inhibition resulted in a significant reduction of nicotine self-administration if injected into the NAc shell but not into the adjacent NAc core. Moreover, as can be observed from the figure, the ventricle is equally distant from the injection sites of the NAc shell and NAc core. Hence, in case of an effect mediated by distal areas or neighboring regions, this would have occurred following injection into both subregions. Bottom, Photos are examples of rats injected with DMSO vehicle (left, right). Center, Example of rat that received aCSF vehicle. To facilitate the visualization of the injector trace, rats were injected with blue ink postmortem. As can be observed, the tissue damage following DMSO or aCSF is comparable.

**Figure 3.**
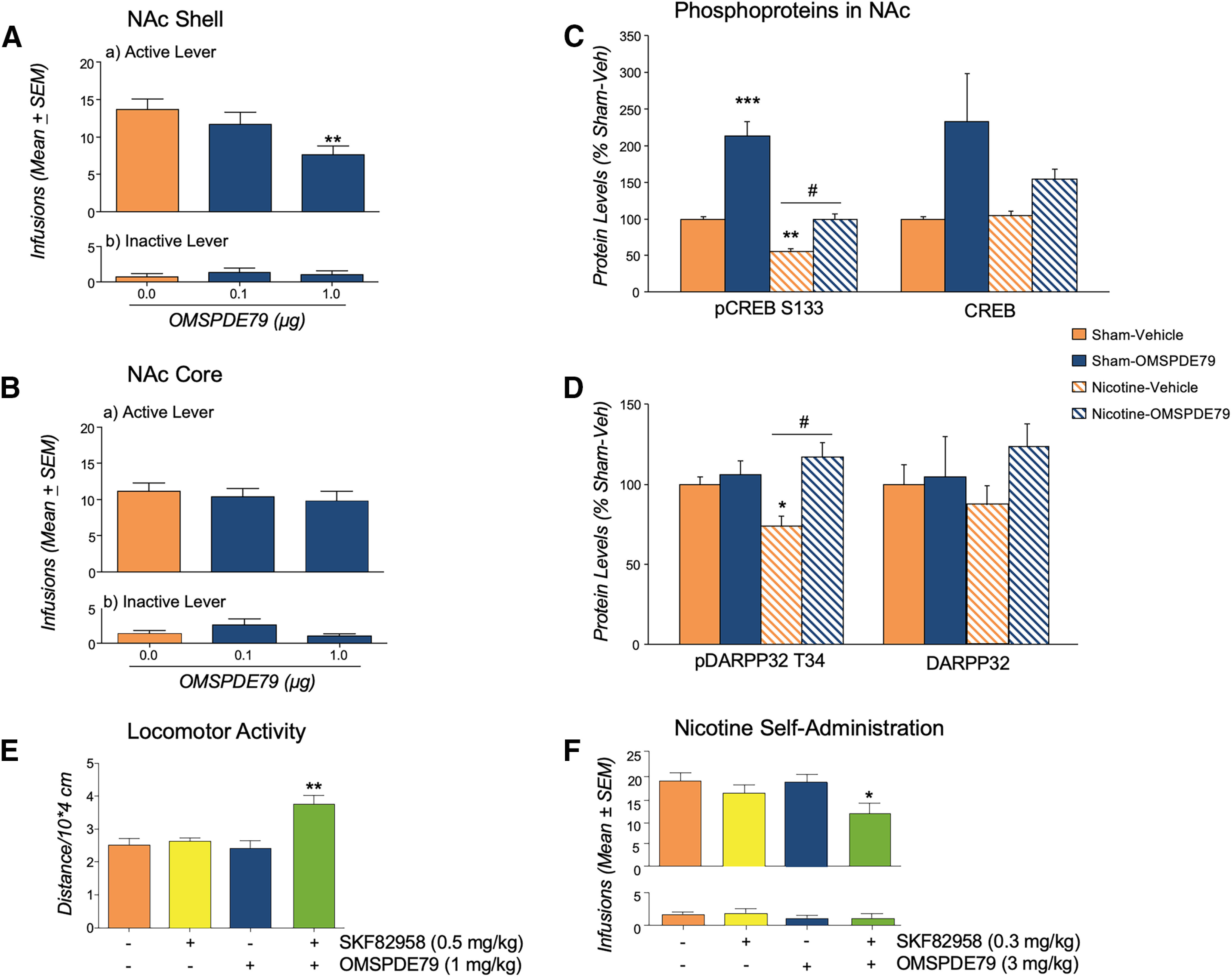
Effect of PDE7i on nicotine self-administration following intra-NAc delivery and on the regulation of D1 receptor-mediated activity. ***A***, Rats (*n* = 8) trained to nicotine self-administation and injected with OMSPDE79 into the NAc shell 10 min before the beginning of the session showed a marked decrease in nicotine intake. ***B***, Following microinjection into the NAc core of a different group of rats (*n* = 10), no effects were detected. Inactive lever responses were not affected by treatments. ***C***, ***D***, Rats (*n* = 5–6/group) subjected to nicotine self-administration training or sham operation were used to evaluate the levels of pCREB/CREB and pDARPP32/DARPP32. Results showed that exposure to nicotine results in adaptive changes to this pathway, suggesting reduced D1-mediated intracellular activities in the NAc. Inhibition of PDE7 enhances PKA-mediated signaling. ***E***, In the open field test, the combination of OMSPDE79 and SKF-82958, but neither drug alone, enhanced locomotor activity over baseline. ***F***, Coadministration of a low dose of OMSPDE79 and SKF-82958, but neither drug alone, reduced nicotine self-administration. ***A–F***, Values represent the mean (± SEM) of nicotine infusions (***A***, ***B***, ***F***), protein levels (***C***, ***D***), or distance traveled (***E***). Significant difference from controls (0.0), *p < 0.01 and **p < 0.001. Significant difference between controls and nicotine plus OMSPDE79, #*p* < 0.05.

Two groups of rats (*n* = 11–12/group) were trained for intravenous nicotine self-administration (FR1 reinforcement schedule) and implanted with bilateral intracranial cannulae aimed at the NAc shell or core. In a within-subject design, the two groups of rats (*n* = 8 and *n* = 10 with correct cannula placement in the NAc shell and core, respectively; Fig. 2) received OMSPDE79 (0, 0.1 and 1.0 μg/0.6 μl) 10 min before the test sessions. As shown in Figure 3*A*, injection of OMSPDE79 into the NAc shell 10 min before the beginning of the session elicited a marked decrease in nicotine intake (*F*_(2,7)_ = 10.32, *p* = 0.0082) with a significant difference from the vehicle at 1.0 μg (Dunnett's, **p < 0.01). As shown in Figure 3*B*, no drug effect was observed following microinjection into the NAc core [*F*_(2,9)_ = 0.31, *p* = 0.74 (NS)]. Inactive lever responses were not affected by treatments either in the shell [*F*_(2,7)_ = 0.315, *p* = 0.74 (NS)] or in the core [*F*_(2,9)_ = 1.52, *p* = 0.23 (NS)]. At completion of the experiment, histologic verification revealed incorrect cannula placement in three rats of the NAc shell group and in two of those in the NAc core group. Data from these rats were not included in the statistical analysis (Fig. 2).

### PDE7 inhibition enhances D1-mediated intracellular signaling in the NAc of rats exposed to nicotine

Rats (*n* = 5–6/group) were trained to nicotine self-administration until a stable lever-pressing baseline was reached. Twenty-four hours after the last self-administration session, rats received either OMSPDE79 or the vehicle. Nicotine naive, sham-operated rats were used as controls. The levels of DARPP-32 and its PKA-dependent phosphorylation at Thr^34^ (pDARPP-32 T^34^) as well as CREB and its PKA-dependent phosphorylation at Ser^133^ (pCREB S^133^) in NAc extracts from both treatment and control rats were quantitated in Western blots using selective antibodies (Carlezon et al., 2005; Beaulieu and Gainetdinov, 2011). As shown in Figure 3*C*,*D*, a two-way ANOVA on data normalized for the respective controls revealed an overall effect of OMSPDE79 (9.0 mg/kg, i.p.) on pCREB (*F*_(1,17)_ = 56.51, *p* = 0.0001) and on CREB (*F*_(1,17)_ = 8.22, *p* = 0.01), as well as on pDARPP32 (*F*_(1,16)_ = 11.20, *p* = 0.0041) but not on DARPP32 [*F*_(1,16)_ = 1.611, *p* = 0.2226 (NS)] in the nuclear fraction of NAc. The ANOVA also revealed a significant reduction of pCREB in the nicotine self-administration group treated with the OMSPDE79 vehicle (*F*_(1,17)_ = 56.97, *p* = 0.0001). Significant increases in pCREB (*F*_(1,17)_ = 10.75. *p* = 0.0044] and pDARPP32 (*F*_(1,16)_ = 6.307, *p* = 0.0231) were observed in nicotine-exposed animals treated with OMSPDE79 (Nic-OMSPDE79) compared with those self-administering nicotine plus PDE7i vehicle. Dunnett's *post hoc* analysis revealed also a significant (**p < 0.01) decrease of CREB phosphorylation in S^133^ in nicotine-exposed rats treated with OMSPDE79 vehicle compared with controls (no nicotine, no PDE7i). Nicotine-induced reduction of pCREB was normalized by OMSPDE79 (#*p* < 0.5). Compared with sham-operated controls, administration of OMSPDE79 significantly increased pCREB expression in nicotine-naive rats (Sham-OMSPDE79; ***p < 0001) but not in nicotine-exposed rats (Nic-OMSPDE79). Dunnett's *post hoc* analysis revealed a significant (*p < 0.05) decrease of DARPP32 phosphorylation in T^34^ in nicotine-exposed rats treated with OMSPDE79 vehicle. The nicotine-induced reduction of pDARPP32 was reversed by OMSPDE79 (##*p* < 0.5).

The D1-PDE7 connection was also evaluated in a behavioral setting. In this case, we determined the impact of PDE7 inhibition on D1 receptor-mediated hyperlocomotion (Schindler and Carmona, 2002). For this purpose, we examined the effects of OMSPDE79 and the D1 receptor agonist SKF-82958 (O'Boyle et al., 1989), alone and in combination, in open field activity (OFA) in the rat. As shown in Figure 3*E*, a two-by-two ANOVA revealed a significant drug interaction on locomotor activity (*F*_(3,12)_ = 8.96, *p* = 0.001), justified by the lack of significant effects of PDE7i (1.0 mg/kg) and D1 agonist (0.5 mg/kg) alone. However, when SKF-82958 and OMSPDE79 were combined, we observed a significant (*p* < 0.01) enhancement of OFA, suggesting potentiation of the D1 receptor activity by PDE7 inhibition.

Furthermore, to directly assess whether potentiation of D1-mediated activity by PDE7i is associated with the attenuation of nicotine consumption, in a new group of rats (*n* = 8) we tested the effect of OMSPDE79 (3.0 mg/kg) and SKF-82958 (0.3 mg/kg) alone and in combination on nicotine self-administration. As shown in Figure 3*F*, a two-by-two ANOVA revealed a significant overall effect of treatment (*F*_(3,28)_ = 3.57, *p* = 0.04) with the compounds showing reduction of nicotine-related lever pressing when given in combination.

### Inhibition of PDE7 attenuates DA firing in the VTA and is devoid of motivational properties

**Figure 4.**
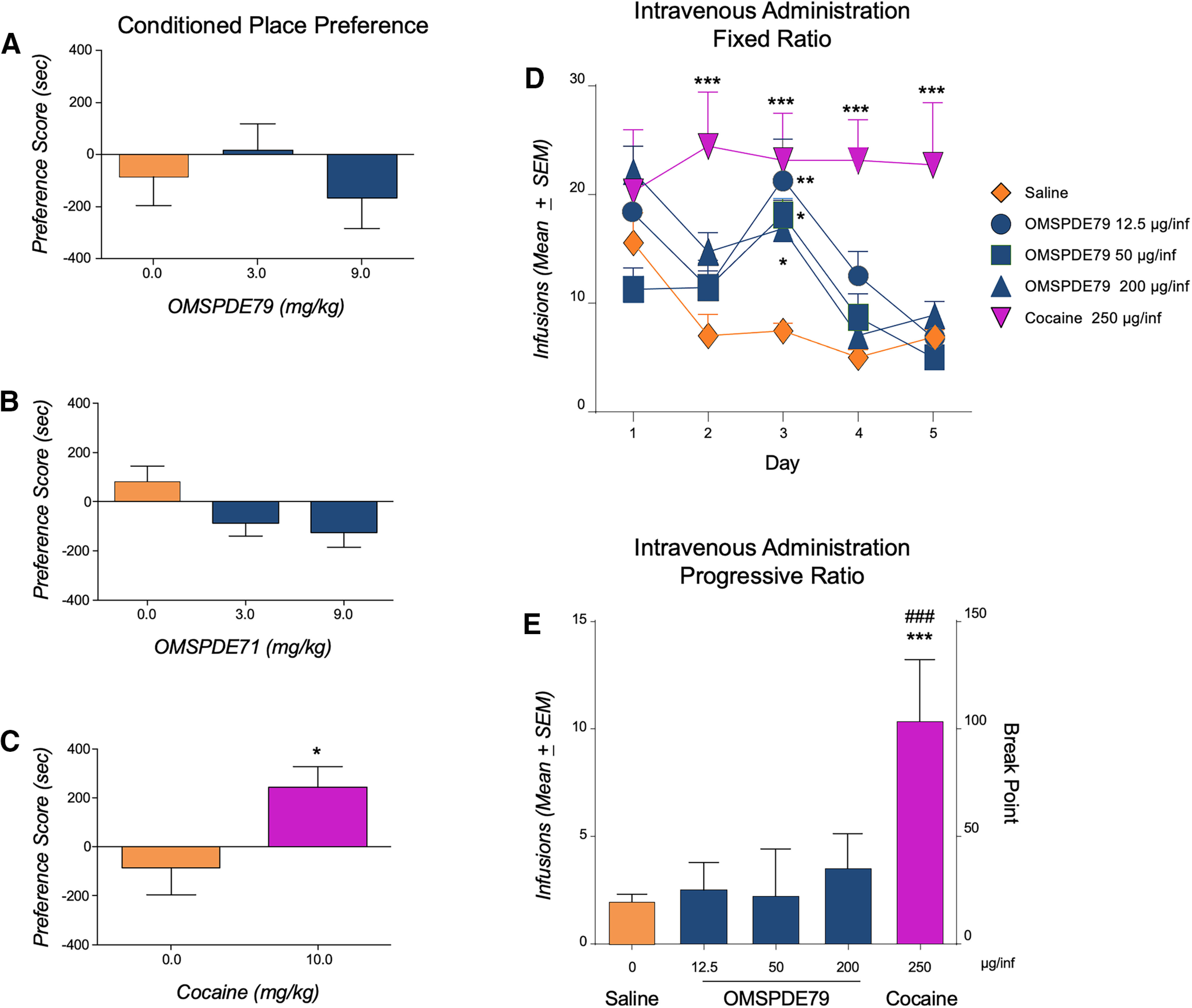
PDE7 inhibition does not cause place preference and is not reinforcing. ***A***, ***B***, Conditionings with OMSPDE79 (***A***) or with OMSPDE71 (***B***) did not lead to development of place preference. ***C***, Cocaine produced a marked expression of conditioned place preference as revealed by the marked increase in the percentage of time spent in the drug-paired compared with the vehicle-paired compartment. ***D***, Different groups of rats (*n* = 7/group) initially trained to FR1 intravenous cocaine self-administration were then switched to intravenous saline, to various doses of OMSPDE79 (12.5, 50, and 200 µg/infusion), or were maintained on cocaine (250 µg/infusion). Lever presses dropped markedly in all groups of rats except the one maintained on cocaine. ***E***, In the same group of rats, after baseline for cocaine was reestablished, operant responding for cocaine, OMSPDE9, and saline was evaluated in a PR paradigm. The break point of OMSPDE7 was indistinguishable from that of saline and was much lower compared with that reached by the cocaine group. ***A–E***, Values represent the mean (± SEM) of preference score (***A***, ***B***, ***C***) or nicotine infusions (***D***, ***E***). Data are expressed as mean ± SEM. Significant difference from saline, *p < 0.05, **p < 0.001, ***p < 0.0001. Significant difference between cocaine and OMSPDE79, ###*p* < 0.001.

In an unbiased conditioned place preference (CPP) design, different groups of rats (*n* = 8/group) were conditioned with OMSPDE79 (0, 3.0 or 9.0 mg/kg), OMSPDE71 (0, 3.0 or 9.0 mg/kg), or with cocaine (10.0 mg/kg). As shown in Figure 4*A–C*, conditionings with OMSPDE79 or OMSPDE71 did not lead to development of place preference [*F*_(2,21)_ = 0.71, *p* = 0.5 (NS) and *F*_(2,21)_ = 1.4, *p* = 0.3 (NS), respectively]. Conversely, cocaine produced a marked expression of CPP that was evidenced by a significant [df(15), *t* = 2.34, *p < 0.05] increase in the percentage of time spent in the drug-paired compared with the vehicle-paired compartment (Fig. 4*C*).

To further explore the motivational properties of PDE7i, we next examined the possibility of having these molecules self-administered intravenously by the animals. In this study, we used OMSPDE79 in a wide range of concentrations (12, 50, and 200 µg/infusion). The selection of doses was guided by the PK profile of OMSPDE79. Specifically, after in intraperitoneal administration of 3 mg/kg of the compound, the maximum concentration (Cmax) was 649 ± 54.8 ng/ml, and the maximum time (Tmax) was 0.3 ± 0.1 h, whereas after intravenous administration of 3 mg/kg the Cmax was 628 ± 81 n/ml, and the Tmax 0.2 ± 0.1 h. In the relapse experiment, PDE7i showed efficacy at intraperitoneal doses as low as 0.3 mg/kg. Considering that in the self-administration experiment the highest dose of PDE7i was 0.2 mg/kg, two infusions would have been sufficient to reach the effective dose of the relapse study. OMSPDE79 was studied because it is more saline soluble than OMSPDE71. We trained a group of rats to self-administer intravenously cocaine (250 µg/infusion) in an FR1 schedule of reinforcement until a stable baseline of responding was achieved (Ator and Griffiths, 2003). At this point, we divided the rats into five groups (*n* = 7/group). Three of them were switched to intravenous self-administration of OMSPDE79 (12, 50 and 200 µg/infusion), and a fourth group was maintained on intravenous cocaine, whereas the fifth one received intravenous saline. All groups were monitored for five consecutive days. Two-way ANOVA showed a significant overall effect of treatment (*F*_(4,30)_ = 5.46, *p* = 0.002) and a significant effect of time (*F*_(4,120)_ = 15.52, *p* = 0.0001), and of treatment per time interaction (*F*_(16,120)_ = 3.6, *p* = 0.000023). The Tukey's *post hoc* test revealed a significant difference from the cocaine and the saline group starting from day 2 (*p* < 0.001). Saline-related responding was lower than OMSPDE79 12.5 (*p < 0.05), OMSPDE79 50 (**p < 0.01), and OMSPDE79 200 (***p < 0.001) on day 3 only, suggesting a progressive drop in lever pressing in all groups compared with cocaine (Fig. 4*D*).

After the experiment, the cocaine baseline was reestablished in all groups of rats. At this point, a second experiment was conducted, and the animals were tested in a PR paradigm (scale 5, 11, 18, 26, 35, 45, 56, 68, 82, 98, 116, 136, 158, 182, 208, 236, 268, and 304). ANOVA displayed a significant effect of treatment (*F*_(4,30)_ = 25.77, *p* = 0.00,001) on the number of infusions obtained. As shown in Figure 4*E*, *post hoc* analysis revealed a significant (***p < 0.0001) difference between the cocaine group and rats responding for saline or OMSPDE79 (###*p* < 0.0001).

**Figure 5.**
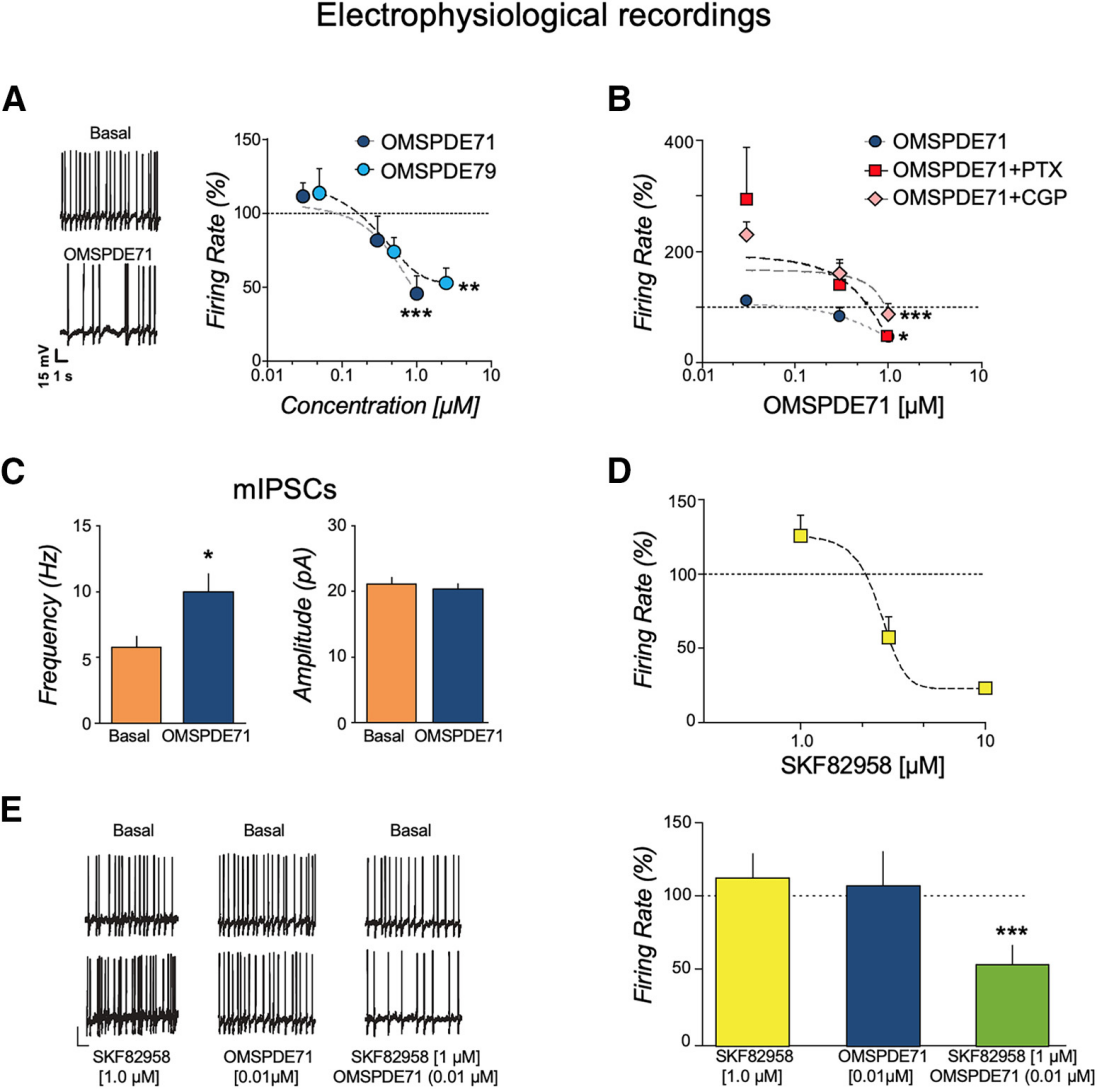
Effect of PDE7 inhibition on VTA dopamine neurons is potentiated by D1 receptor activation. ***A***, OMSPDE79 (*n* = 15) and OMSPDE71 (*n* = 15) significantly decreased spontaneous activity of DA neurons. Left, Current-clamp recording from a DA neuron in the absence (basal) and in the presence of OMSPDE71 (1 μm). Right, Dose–response curves for averaged effects of the two PDE7i. ***B***, Graph summarizing the dose–response effects of OMSPDE71 on DA neuronal frequency in the presence of either the GABA_A_ antagonist picrotoxin (*n* = 6) or the GABA_B_ antagonist CGP35348 (*n* = 6), respectively. ***C***, Miniature IPSCs recorded under voltage-clamp mode (V_m_ = −70 mV) from DA neurons (*n* = 11). As shown, acute application of OMSPDE71 (1 μm, 5 min) increases the frequency without affecting the amplitude. ***D***, Dose–response curve for the effects of the D1 agonist SKF-82958 on spontaneous activity of DA neurons (*n* = 10). ***E***, Left, Current-clamp recordings from dopamine neurons in the absence (basal) and in the presence of ineffective concentrations of either SKF-82958 (*n* = 8) or OMSPDE71 (*n* = 8) alone or when coapplied (OMSPDE71+SKF-82958; *n* = 13). Right, Bar graph summarizing the effects. Data are expressed as mean ± SEM. Significant difference from controls, *p < 0.05, **p < 0.001, ***p < 0.0001.

We next assessed the effect of PDE7 inhibition on DA cell firing from brain slices containing the posterior VTA by using whole-cell patch-clamp recordings. As shown in Figure 5*A*, acute bath application of OMSPDE79 or OMSPDE71 (0.03, 0.3 and 1 μm for 5 min) significantly decreased the spontaneous activity of VTA DA neurons in a dose-dependent fashion (OMSPDE79: one-way ANOVA, *F*_(3,42)_ = 5.67, *n* = 15, *p* = 0.002; OMSPDE71: one-way ANOVA, *F*_(3,42)_ = 7.92, *n* = 15, *p* = 0.00,027). To evaluate whether PDE7 inhibition was because of enhanced presynaptic GABA transmission, we tested the effect of OMSPDE71 in the presence of either the GABA_A_ antagonist picrotoxin (PTX) or the GABA_B_ CGP35348 (CGP), respectively, each at a concentration of (100 μm). ANOVA revealed a significant overall effect of treatments with both PTX (*F*_(1,38)_ = 7.98, n = 6, *p* = 0.01) and CGP35348 (*F*_(1,36)_ = 20.24, *n* = 6, *p* = 0.0003). However, as revealed by *post hoc* analysis following treatment with PTX, the inhibition of firing rate by OMSPDE71 was still present at the highest concentration of PDE7i **(**Fig. 5*B*). Conversely, when we applied OMSPDE71 in the presence of the GABA_B_ antagonist CGP35348, it was no longer able to inhibit the firing rate of DA neurons (Fig. 5*B*). Altogether these data suggest that both postsynaptic GABA_B_ and GABA_A_ receptors might contribute to the effects induced by OMSPDE71 on DA neuronal activity. One possibility is that the effects observed may be, at least in part, linked to the ability of PDE7i to enhance GABA release from presynaptic terminals. To test this notion, we collected GABA_A_-mediated miniature inhibitory postsynaptic currents (mIPSCs) under a voltage-clamp mode in the presence of lidocaine (500 μm) and OMSPDE71. As shown in Figure 5*C*, we observed an increased frequency (by 72%; paired *t* test, n = 11, *t* = 2.67, *p* < 0.01) with no change in the amplitude [paired *t* test, *n* = 11, *t* = 0.79 (NS)], thus indicating that PDE7 inhibition increases the probability of GABA release in the VTA from presynaptic terminals. Interestingly, when GABA_A_ and GABA_B_ receptors were blocked, low doses of the PDE7 inhibitor excited VTA DA neuronal activity.

We also assessed the effects of OMSPDE71 in combination with the D1 receptor agonist SKF-82958 on the DA firing rate. Like OMSPDE71, ANOVA showed that acute application of SKF-82958 (1.0, 3.0 and 10 μm, 5 min) significantly (*F*_(3,21)_ = 5.34, *n* = 10, *p* = 0.016) decreased the spontaneous activity of VTA DA neurons (Fig. 5*D*). Remarkably, simultaneous application of ineffective concentrations of each compound (30 nm OMSPDE71 and 1 μm SKF-82958) significantly (*F*_(2,34)_ = 9.32, *p* = 0.0005) reduced the spontaneous firing in VTA DA cells (Fig. 5*E*).

### Inhibition of PDE7 prevents reinstatement of stress- or cue-induced nicotine seeking

**Figure 6.**
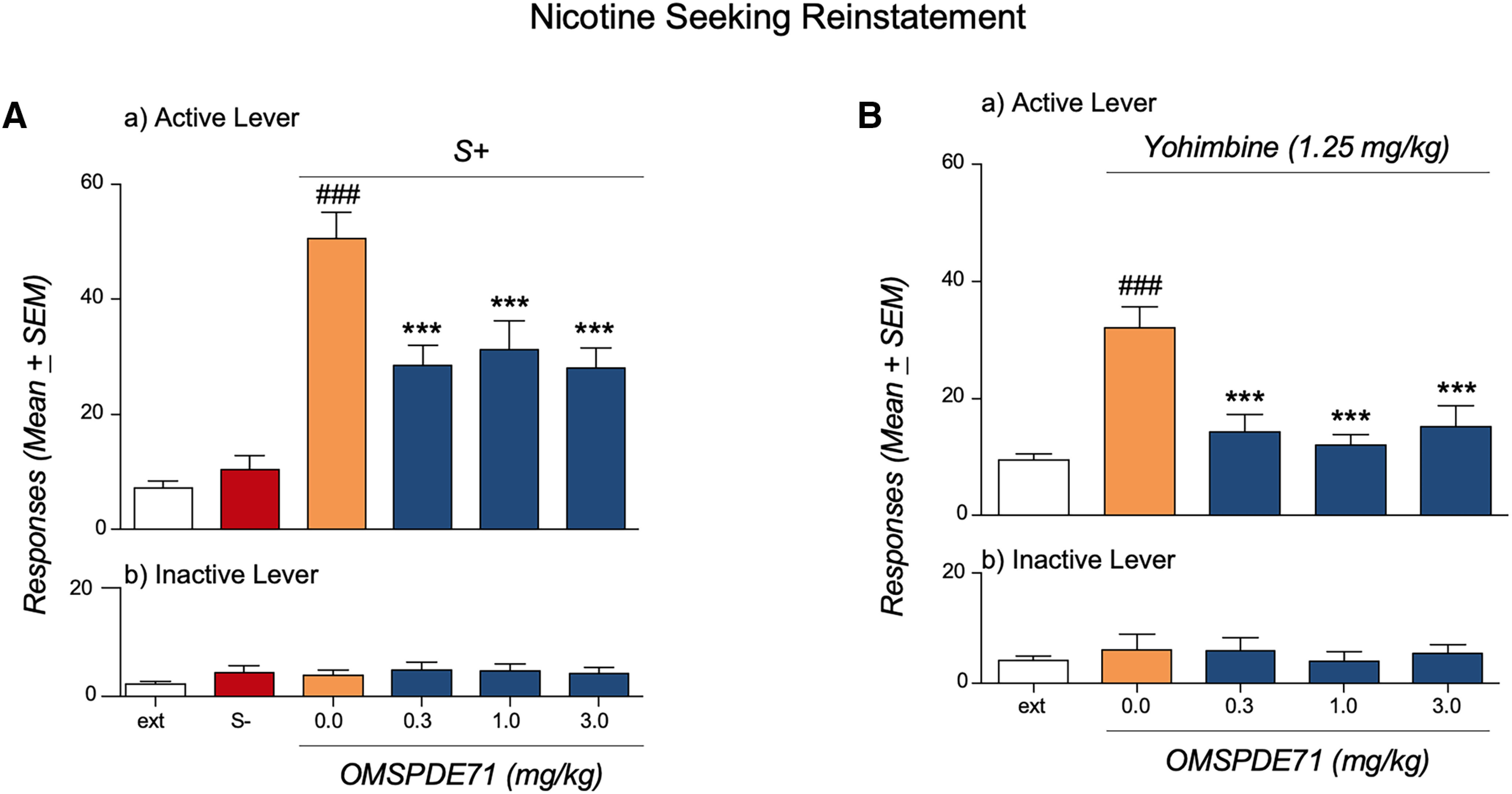
PDE7 inhibition attenuates reinstatement of nicotine seeking. ***A***, For cue-induced reinstatement of nicotine seeking, rats (*n* = 15) previously trained to a self-administration/discrimination procedure and then subjected to an extinction phase were treated with OMSPDE71. Compared with extinction (ext), reintroduction of nicotine (S+)-associated but not saline (S-)-associated cues elicited reinstatement. Treatment with OMSPDE71 significantly attenuated it. ***B***, For yohimbine-induced reinstatement of nicotine seeking, rats (*n* = 15) previously trained to nicotine self-administration and then subjected to an extinction phase were treated with OMSPDE71 followed by yohimbine. Responses were recorded for 1 h. Compared with extinction, administration of yohimbine significantly reinstated responding at the previously nicotine-associated lever. OMSPDE71 significantly attenuated yohimbine effects on nicotine seeking. Responses at the inactive lever were always very low and not affected by treatments. ***A***, ***B***, Values represent the mean (±SEM) number of the active lever responses (a) and responses at inactive lever (b). Significant difference from controls (0.0) and from extinction (ext); ***p < 0.001 and ###*p* < 0.001, respectively.

Rats (*n* = 15) were trained to self-administer nicotine and to discriminate between nicotine and saline. A distinct discriminative stimulus (S^+^) was present during availability of nicotine, whereas a different discriminative stimulus (S^−^) was present during availability of saline (i.e., nonreward). During these sessions, the number of nicotine-reinforced responses was significantly greater than responses during the saline session (On the last discrimination day, responses were 10.1 ± 0.5 and 1.8 ± 0.3 for nicotine and saline, respectively.). Following this discrimination phase, rats were subjected to daily extinction sessions during which nicotine, saline, and the corresponding contextual stimuli were withheld. Responding decreased progressively during this phase, and the animals reached an extinction criterion of ≤10 responses per hour (Fig. 6*A*). In the reinstatement test, the rats were tested for recovery of responding in the presence first of the S^−^ followed by the S^+^. These reinstatement tests were conducted under stimulus conditions identical to those during the self-administration/conditioning session, except that nicotine (or saline) was not made available. ANOVA revealed that compared with extinction, reintroduction of nicotine-associated (S+) but not saline-associated (S−) cues elicited reinstatement as revealed by increased responding at the previously active nicotine-paired lever (*F*_(2,14)_ = 90.78, *p* = 0,00,001) but not at the inactive lever [*F*_(2,14)_ = 1.79, *p* = 0.2 (NS)]. *Post hoc* Dunnett's comparisons demonstrated a significant difference between extinction and S+ (*p* < 0.01). Treatment with OMSPDE71, given in a within-subject Latin square counterbalanced design (treatment interval 3 d), significantly attenuated seeking responses (*F*_(3,14)_ = 18.23, *p* < 0.00,004) with an observed effect at all doses tested (Dunnett's; ***p < 0.001). Responses at the inactive lever were not affected by treatment [*F*_(3,14)_ = 1.84, *p* = 0.18 (NS)].

A different group of rats (*n* = 15) was trained to nicotine self-administration until a stable (10.8 ± 0.95) baseline of responding was achieved. Twenty-four hours later, the rats were subjected to daily extinction sessions during which nicotine was no longer available. Responding decreased progressively during this phase, and after 10 d, extinction levels were below 10 responses per hour (Fig. 6*B*). At this point, the reinstatement test took place. Rats were treated in a within-subject Latin square counterbalanced design with OMSPDE71 followed by yohimbine injection 30 min later. Relapse tests took place every 3 or 4 d, and during intervals, rats were subjected to extinction sessions. In the relapse test, responses were recorded for 1 h. Compared with extinction, administration of yohimbine significantly reinstated responding at the previously nicotine-associated lever [df(14), *t* = 6.219, ###*p* = 0.0001]. No effects were observed at the inactive control lever [df(14), *t* = 0,15, *p* = 0.88 (NS)]. ANOVA revealed that OMSPDE71 significantly attenuated yohimbine effects on nicotine seeking (*F*_(3,14)_ = 9.06, *p* = 0.0014). *Post hoc* Dunnett's comparisons showed an effect of PDE7i at all doses tested (***p < 0.001). Responses at the inactive lever were not affected by yohimbine or OMSPDE71 treatments [*F*_(3,14)_ = 0.94, *p* = 0.45 (NS)].

**Figure 7.**
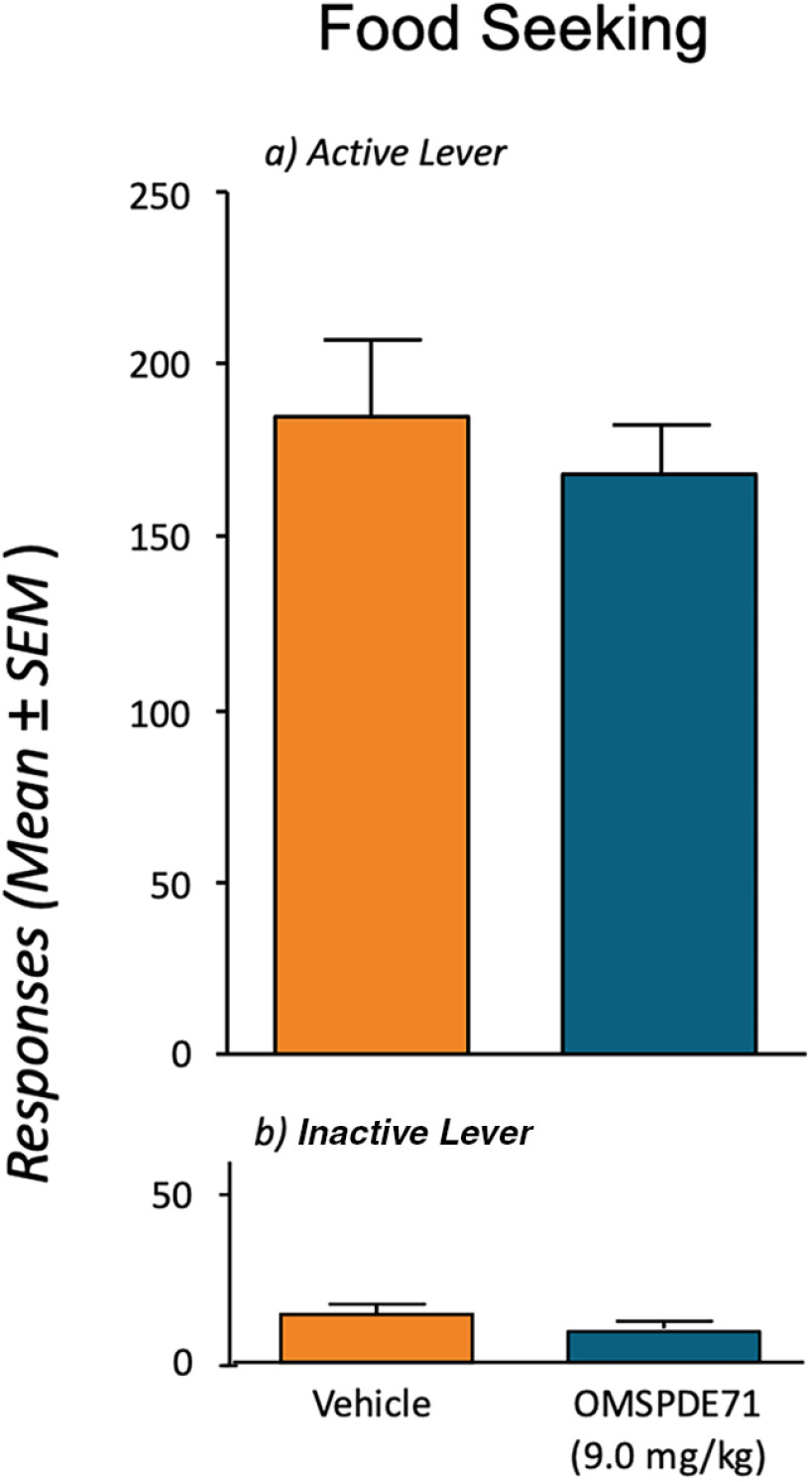
PDE7 inhibition does not affect extinction responding in food trained rats. Rats were trained to operant food self-administration and then subjected to an extinction session during which lever presses were no longer reinforced. Lever presses in the absence of food delivery were considered a measure of seeking response. Thirty minutes before this extinction session, rats were treated with intraperitoneal administration of OMSPDE71 (9 mg/kg) or its vehicle, and operant responding was recorded for 30 min.

To control for the specificity of the effect of OMSPDE71 on reinstatement, we trained an additional group of rats to food pellet self-administration. On the first extinction day, half of the animals were treated with OMSPDE71 (9.0 mg/kg), and the other half received the vehicle. In the control group, a significantly high level of responding was detected during this first extinction day, reflecting animals' motivation for food. OMSPDE71 did not modify this effect (Fig. 7), suggesting that the effect of OMSPDE71 on nicotine seeking does not depend on impairment of locomotor function or on general inhibition of motivation during the relapse session. Similar results were also observed with OMSPDE79 (data not shown).

## Discussion

In the present study, we used two different small-molecule inhibitors of PDE7—OMSPDE79 and OMSPDE71. These molecules are potent and selective inhibitors of both PDE7A and PDE7B. Their IC_50_ against PDE7A or PDE7 B are <20 nm, and the selectivity against the other PDEs tested is at least 100-fold. Both compounds cross the blood–brain barrier, and their half-lives in rodents range from 0.7 to .3 h. When the effects of OMSPDE79 and OMSPDE71 on intravenous nicotine self-administration were tested, we observed a significant drop in the motivation for the drug, evidenced by the reduction in the number of infusions under both FR and PR contingencies. The effect was selective given that at the same doses the PDE7 inhibitors did not change the operant response for food, nor did they affect pressing at the inactive control lever. It is noteworthy that the effects of OMSPDE79 and OMSPDE71 were observed against nicotine given intravenously over a range of doses, which have been shown to be affected by bupropion and varenicline, medications already approved for smoking cessation in humans (Rauhut et al., 2003; Hall et al., 2015)

The corticomesolimbic DA system that projects from the VTA to the NAc plays a major role in mediating the motivational properties of nicotine (Corrigall et al., 1992, 1994; Pontieri et al., 1996; Picciotto and Kenny, 2013). Acute nicotine administration, acting on nicotinic acetylcholine receptors (AChRs) in the VTA, enhances glutamatergic transmission and simultaneously desensitizes GABA-releasing neurons (Mansvelder and McGehee, 2000; Mansvelder et al., 2002), increasing the phasic burst firing of DA neurons and enhancing the extracellular DA levels preferentially in the NAc shell (Pontieri et al., 1996; Zhang et al., 2009). This action appears to be facilitated by nicotine-induced inhibition of striatal DA release through activation of AChRs on striatal DAergic terminals (Wang et al., 2014), with subsequent accumulation of DA vesicle pools and enhanced contrast between DA release evoked by phasic and tonic firing (Zhou et al., 2001; Rice and Cragg, 2004; Zhang and Sulzer, 2004). The increase in DA transmission in target areas is essential for reward learning and acquisition of nicotine self-administration (Sulzer, 2011). Both PDE7A and PDE7B are expressed in the NAc (Miró et al., 2001; Reyes-Irisarri et al., 2005; Johansson et al., 2012)

We then examined the impact of PDE7 inhibition in this region through operant intravenous nicotine self-administration. Rats were microinjected with OMSPDE79 into the NAc shell and NAc core, and operant responding for nicotine was monitored. We observed a marked reduction in nicotine self-administration following drug delivery to the NAc shell but not to the NAc core.

Following a history of nicotine self-administration, adaptive changes in the mesolimbic transmission occur. These led to deficiencies in extracellular DA in the NAc, measured 24 h from the last nicotine infusion (Rahman et al., 2004). Similar findings have been documented in animals exposed to passive intermittent administration of nicotine, known to unmask a marked hypo-DAergic state characterized by decreased DA release in the NAc and reward deficiency (Epping-Jordan et al., 1998; Kenny and Markou, 2005; Domino et al., 2009; Natividad et al., 2010; Zhang et al., 2012; Carcoba et al., 2017). Accordingly, we hypothesized that PDE7 inhibition in the NAc shell reduced the motivation for nicotine by reestablishing normal DA transmission in this region. In fact, in the striatum, PDE7 enzymes are highly expressed in both D1- and D2-positive cells, and >50% of striatoaccumbal medium spiny neurons (MSN) express high levels of the PDE7B isoform (Sasaki et al., 2004; de Gortari and Mengod, 2010). Modulation of PDE7 enzymatic activity might affect the function of both the direct and the indirect pathways in these GABAergic cells (Kravitz et al., 2010; Gutierrez et al., 2011; Xia et al., 2011). More specifically, as already observed for PDE10, PDE7 inhibition could enhance cAMP-PKA-dependent signaling downstream of D1 receptors that will increase DA-mediated neurotransmission at the postsynaptic level, counteracting the hypo-DAergic state that occurs during abstinence from nicotine (Rahman et al., 2004; Domino et al., 2009; Sotty et al., 2009; Natividad et al., 2010; Zhang et al., 2012; Carcoba et al., 2017).

To test this hypothesis, animals subjected to nicotine self-administration were used to determine the levels of cAMP-regulated phosphoproteins DARPP-32 and CREB, two postsynaptic markers linked to DA transmission in MSN (Kebabian and Greengard, 1971; Carlezon et al., 2005; Beaulieu and Gainetdinov, 2011).

We found that chronic exposure to nicotine reduced the levels of DARPP-32 and CREB phosphorylated forms, indicating reduction of PKA-dependent activity after 24 h of nicotine abstinence. Notably, this time point corresponds to the time at which, in the self-administration experiment, rats would have returned to nicotine self-administration. The presence of the PDE7i reversed this condition, suggesting that PDE7 inhibition restores intracellular signaling, likely through D1 neurotransmission. Importantly, we also detected a marked increase in CREB and pCREB levels in sham-operated nicotine-naive rats but not in animals with a history of nicotine self-administration. This is consistent with the notion that inhibition of PDE7 enhances PKA-mediated signaling and that exposure to nicotine results in adaptive changes to this pathway, contributing to reduced D1-mediated intracellular activities in the NAc. Interestingly, in nicotine-naive rats, PDE7i did not affect DARPP-32 signaling, suggesting that PDE7 inhibition and the subsequent increase in PKA activity may differentially affect the DARP32 and CREB pathways (Heckman et al., 2018).

To provide the functional correlates of these biochemical findings, we evaluated the impact of PDE7 inhibition on D1 receptor-mediated hyperlocomotion (Schindler and Carmona, 2002). For this purpose, we examined the effects of OMSPDE79 and the D1 receptor agonist SKF-82958 (O'Boyle et al., 1989), alone or in combination, in OFA in rats. Consistent with the hypothesis that PDE7 inhibition potentiates D1-mediated activity, we observed that ineffective doses of OMSPDE79 and SKF-82958 led to a significant increase of locomotor activity when combined. To directly assess whether potentiation of D1-mediated activity by PDE7i is associated with the attenuation of nicotine consumption, we tested the effects of OMSPDE79 and SKF-82958 alone or in combination on nicotine self-administration. Consistent with the locomotor activity data, we registered a significant attenuation of nicotine self-administration following coadministration of SKF-82958 and OMSPDE79: At these doses, the drugs were not effective if given individually.

Potentiation of D1 activity produces reward-related learning and is sufficient to elicit a CPP response in rodents (Abrahams et al., 1998). Moreover, selective D1 agonists, like SKF-82958, can be self-administered by rats and monkeys trained to administer cocaine (Self and Stein, 1992; Grech et al., 1996; Weed et al., 1997). Given that in nicotine-naive rats, PDE7i increased both the D1-related CREB function in the NAc and the behavioral effects of SKF-82958, we evaluated OMSPDE79 and OMSPDE71 in CPP for rewarding effects. We also examined whether OMSPDE79 maintains operant self-administration in rats previously trained to cocaine self-administration. In both experiments the compounds showed no effects, demonstrating that the PDE7 inhibitors lack reinforcing properties and are not voluntarily self-administered.

In addition to NAc, PDE7 is present in the VTA where potentiation of cAMP-dependent PKA signaling has been associated with increased presynaptic GABA release and enhanced activation of GABA_A_ and GABA_B_ receptors, leading to inhibition of VTA DA neurons (Cameron and Williams, 1993; Bonci and Williams, 1997). It is possible that the lack of reinforcing effects by the PDE7 inhibitors is associated with their ability to attenuate DA activity in the VTA. To evaluate this possibility, we assessed the effect of PDE7 inhibition on DA cell firing from brain slices containing the posterior VTA using whole-cell patch-clamp recordings. We found that OMSPDE79 or OMSPDE71 decreased the spontaneous activity of VTA DA neurons through increased probability of presynaptic GABA release and subsequent stimulation of both GABA and GABA_B_ receptors. In fact, the effect of PDE7 inhibitors on DA cell firing was completely prevented by coadministration of GABA_A_ (picrotoxin) and GABA_B_ (CGP35348) antagonists. Interestingly, when either GABA_B_ or GABA_A_ receptors were blocked, low doses of the PDE7 inhibitor excited VTA DA neuronal activity. This finding suggests that potentiation of cAMP signaling in the VTA may enhance glutamatergic excitatory transmission, which can be revealed by blocking the inhibitory GABA activity. This observation is consistent with earlier work showing that enhanced cAMP-mediated transmission facilitates presynaptic glutamate release (Chavez-Noriega and Stevens, 1994). Specific studies are needed to further explore the mechanisms by which activation of the cAMP-PKA pathway enhances presynaptic neurotransmission in the VTA.

D1 receptors in the VTA are localized on the terminals of afferent GABA neurons. There they facilitate presynaptic GABA transmission, which, through GABA_B_-mediated mechanisms, can control mesolimbic DA cell firing (Cameron and Williams, 1993). Hence, we predicted that like in the NAc shell, the effect of PDE7 inhibition in the VTA was mediated by potentiation of D1 receptor-mediated activity. Indeed, similar to OMSPDE71, the D1 receptor agonist SKF-82958 decreased the spontaneous activity of the VTA DA neurons. Low doses of each of the two compounds individually were ineffective, but their inhibitory effect on DA firing was reestablished following their combined administration. These findings provide evidence for reduced activity of VTA DA cells from PDE7 inhibition, which is mediated by potentiation of D1 receptor intracellular signaling and the subsequent increase in presynaptic GABA release.

Based on our results, it is tempting to speculate that PDE7 inhibitors attenuate the motivation for nicotine through potentiation of DA transmission in the accumbens via potentiation of D1 receptor-mediated intracellular signaling, whereas they have low intrinsic motivational value because of their ability to blunt the electrical activity of DA neurons in the VTA.

Most people attempting to quit smoking on their own relapse within the first several weeks of abstinence (Ward et al., 1997; Shiffman, 2006). Environmental cues associated with previous use of nicotine is a major determinant of relapse (Pickens et al., 2011; Stoker and Markou, 2015). Another major factor in nicotine relapse is stress and anxiety (Bruijnzeel, 2012). Preclinical studies have shown that yohimbine, an α-2 adrenoreceptor antagonist that invigorates responding to somatosensory stimuli associated with drug effects and acts via complex mechanisms involving both stress responses and recruitment of dopamine transmission, induces robust nicotine seeking in rats and craving in humans (Marinelli et al., 2007; Umhau et al., 2011; Feltenstein et al., 2012; Chen et al., 2015). To further assess the therapeutic potential of PDE7 inhibitors, we evaluated their effects on reinstatement of nicotine seeking elicited by cues or by yohimbine administration. Results revealed a remarkable efficacy of OMSPDE71 and OMSPDE79 each in preventing reinstatement of drug seeking. Reduction of lever pressing was specific in that the PDE7 inhibitors did not modify operant responding in food-trained rats tested during the first extinction day. Notably, PDE7 inhibitors prevented the reinstatement of drug seeking at much lower doses compared with those needed to reduce nicotine intake. This raise the possibility that distinct mechanisms may be involved in mediating these two pharmacological actions of OMSPDE71 and OMSPDE79. A tempting hypothesis is that relapse prevention by these drugs is mediated by enzyme inhibition in the VTA, whereas, as shown here, the effects on nicotine self-administration are mediated by PDE7 inhibition into the NAc shell. This hypothesis is supported by studies showing that disinhibition of DA neurons in the VTA occurs during reinstatement (Mahler et al., 2014). In addition, fluctuations in DA transmission in this area modulate drug intake and reinstatement of drug seeking through activation of different neuronal ensembles projecting to the accumbens (Liu et al., 2020).

In summary, our findings demonstrate that PDE7 inhibition leads to attenuation of nicotine intake and relapse under operant self-administration conditions. We have also shown that PDE7 inhibition enhances postsynaptic D1-mediated transmission in mesolimbic regions.

Preclinical and clinical studies have shown that stimulation of D1 receptors by selective agonists attenuates the motivation for addictive substances in laboratory animals and in humans (Haney et al., 1998, 1999; Edwards et al., 2007). Unfortunately, side effects associated with the use of D1 agonists have hampered the development of this class of compounds for the treatment of addiction (Rascol et al., 2001). We provide evidence that PDE7 inhibitors may offer an alternative therapeutic approach to drug dependency through mechanisms involving potentiation of intracellular signaling associated with activation of D1 receptors without apparent abuse liability. PDE7 inhibition may also result in reduction of D2-mediated transmission, which may represent another mechanism contributing to the effects of the PDE7 inhibitors on nicotine. Future studies will have to be conducted to explore this possibility.

It should be noted that most drugs of abuse act through modulation of the mesolimbic DA transmission and lead to its dysregulation following protracted exposure (Volkow et al., 2007). It is tempting to speculate that PDE7 inhibition may attenuate this dysregulation not only for nicotine but for other drugs of abuse as well.

Our findings support the clinical exploration of the therapeutic potential of PDE7 inhibitors in humans for nicotine dependence.
